# Genome-Wide Association Study and Candidate Gene Mining of Husk Number Trait in Maize

**DOI:** 10.3390/ijms26073437

**Published:** 2025-04-07

**Authors:** Yancui Wang, Shukai Wang, Dusheng Lu, Ming Chen, Baokun Li, Zhenhong Li, Haixiao Su, Jing Sun, Pingping Xu, Cuixia Chen

**Affiliations:** College of Agronomy, Shandong Agricultural University, Tai’an 271018, China; wyc5515@163.com (Y.W.); sdauwangshukai@163.com (S.W.); ldusheng@163.com (D.L.); chming_11@126.com (M.C.); b1774562702@163.com (B.L.); lzh980525@163.com (Z.L.); suhaixiao2019@163.com (H.S.); sunjing010525@163.com (J.S.); 18706388706@163.com (P.X.)

**Keywords:** maize (*Zea mays* L.), husk number, GWAS, favorable allele, dehydration rate

## Abstract

Husk number (HN) trait is an important factor affecting maize kernel dehydration rate after the physiological maturity stage. In general, a reasonable reduction in HN is a key target sought for breeding maize varieties that are suitable for mechanized harvesting. In this study, the HN of a maize natural population panel containing 232 inbred lines was analyzed, and the results showed a broad-sense heritability of 0.89, along with a wide range of phenotypic variation. With the best linear unbiased prediction (BLUP) values across the three environments, a genome-wide association study (GWAS) was conducted using 995,106 single-nucleotide polymorphism (SNP) markers. A total of 16 SNPs significantly associated with HN were identified by the mixed linear model and general linear model using the TASSEL 5.0 software program. A local linkage disequilibrium (LD) study was performed to infer the candidate interval around the lead SNPs. A total of 19 functionally annotated genes were identified. The candidate genes were divided into multiple functional types, including transcriptional regulation, signal transduction, and metabolic and cellular transport. These results provide hints for the understanding of the genetic basis of the HN trait and for the breeding of maize varieties with fewer HN and faster dehydration rate.

## 1. Introduction

Maize (*Zea mays* L.) is one of the most widely cultivated crops in the world and serves as an important source of food, feed, and industrial material [1]. Maize husk is a natural protective barrier enwrapping the ear, originating from the husk primordium at the internodes of the ear stalk [2]. The husks play a crucial role in maintaining optimal temperature and humidity levels for kernel growth, preventing poor grain filling from heat loss or water deficiency [3,4,5], and act as a barrier that not only reduces the risk of pathogen infection but also prevents pests and birds from damaging the ear [6,7,8]. In addition, the husks can temporarily store nutrients coming from other organs and transport them to the ear, and they are also capable of both C3 and C4 photosynthesis, therefore accumulating photosynthetic products by themselves [9,10].

However, excessive husk coverage can hinder moisture dissipation from the kernel after physiological maturity, therefore slowing down the kernel dehydration. Husk-related traits in maize such as husk number (HN), length, width, thickness, and tightness, are important factors influencing the kernel dehydration rate [9,11]. Among these, HN is a key trait that negatively correlates with the kernel dehydration rate [12]. The HN in modern maize varieties has decreased to 9 compared with 14 in old varieties in America [13]. Li et al. even investigated the effects of removing husks on the grain yield and dehydrating rate, and the results indicated that as more husks were removed, the kernel dehydration became faster [12]. Previous studies examined 508 maize inbred lines, and the results showed that the HN ranged from 5 to 20, suggesting significant phenotypic variation in HN and great potential for the genetic improvement of the HN trait [11,14].

The HN is a complex quantitative trait controlled by multiple quantitative trait loci (QTL) with minor effects in maize. A number of studies have used genome-wide association studies (GWAS) and QTL mapping to identify QTL and single-nucleotide polymorphisms (SNPs) related to HN. Zhang et al. identified a total of 7 QTLs for HN on chromosomes 1, 2, 3, 4, 6, and 9 [15]. Among these, the physical interval of *qHN4* was 0.89 Mb and contained 7 candidate genes, which was detected across three different environments. Zhang et al. studied a BC_2_F_8_ population derived from the maize inbred line Mo17 and teosinte X26-4, and found that two HN-associated QTLs (*qHN-1-1* and *qHN-1-2*) each explained 8.9% of the phenotypic variation [16]. Zhou et al. performed a QTL analysis on 204 recombinant inbred lines derived from DH4866 and T877 and identified one QTL on each of chromosomes 1, 3, 6, and 7, among which *qHN7* showed the largest effect, explaining 19.6% of phenotypic variation [17]. Through fine mapping, they narrowed down *qHN7* to a physical interval of 721.1 kb and predicted four candidate genes. With the development of high-throughput sequencing technology, GWAS based on linkage disequilibrium (LD) has emerged as a powerful approach for dissecting the genetic bases of complex traits [18,19,20]. Zhou et al. used 253 maize inbred lines and the Maize SNP3 K Bead-chip to conduct a GWAS for HN. They identified 24 SNPs that were significantly associated with HN (−log_10_(*P*) > 3), among which 8 SNPs were consistently detected across three different environments [14]. Cui et al. utilized 508 maize inbred lines and 543,641 SNPs to detect associated SNPs for husk traits [11]. They identified 5 and 9 SNPs significantly associated with HN using mixed linear models (MLMs) and general linear models (GLMs), respectively. Wang et al. revealed that a 10 bp natural variant in the 3′UTR of *Zea methyltransferase2* (*ZMET2*) was associated with HN in maize [21]. *ZMET2* encodes a DNA methyltransferase, and the HN of *zmet2* mutants significantly increased.

In this study, GWAS was performed for the HN trait in the association panel of 232 maize inbred lines with 995,106 SNPs. Genes within the same LD block of the significantly associated SNPs were further screened to identify potential candidate genes affecting HN. This study aimed to identify HN-related candidate genes and provide new resources for understanding the genetic basis of husk development.

## 2. Results

### 2.1. Phenotypic Variations in Husk Number (HN)

**Figure 1:**
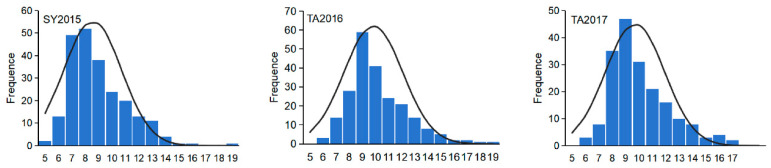
Frequency distribution of husk number. SY2015: Sanya in 2015; TA2016: Taian in 2016; TA2017: Taian in 2017.

The association panel in this study was planted in three environments in Sanya and Taian from 2015 to 2017 (SY2015, TA2016, and TA2017). The HN ranged from 4.80 to 19.00, 6.00 to 18.33, and 5.50 to 16.75, with mean values of 8.56, 9.85, and 9.70 in the three environments, respectively (Table 1), indicating abundant phenotypic variation in the HN trait. The variation in HN follows a normal distribution (Figure 1), with the skewness ranging from 0.94 to 1.15 and kurtosis ranging from 0.63 to 2.01 in different environments, and this is consistent with the characteristics of quantitative traits. High broad-sense heritability (*H*^2^ = 88.62%) was observed for the HN, suggesting that the HN trait is mainly controlled by genetic factors and suitable for further association analysis.

Adopting the classification by Yang et al. [22], the association panel was categorized into four subpopulations: TST, NSS, SS, and Mixed (Table S1). SS and NSS germplasms originate from temperate regions, and TST germplasms from tropical and subtropical areas, with the remaining inbred lines being the Mixed subpopulation. To investigate the impact of population structure on the HN trait, we used phenotypic best linear unbiased prediction (BLUP) values to analyze the phenotypic variation among these four subpopulations. The results indicate that the average husk number in the TST subpopulation was higher than those in NSS and SS (Figure S1), suggesting that maize inbred lines from tropical or subtropical regions tend to have higher husk number.

### 2.2. Genome-Wide Association Analysis (GWAS) and Mining the Favorable Alleles

**Figure 2:**
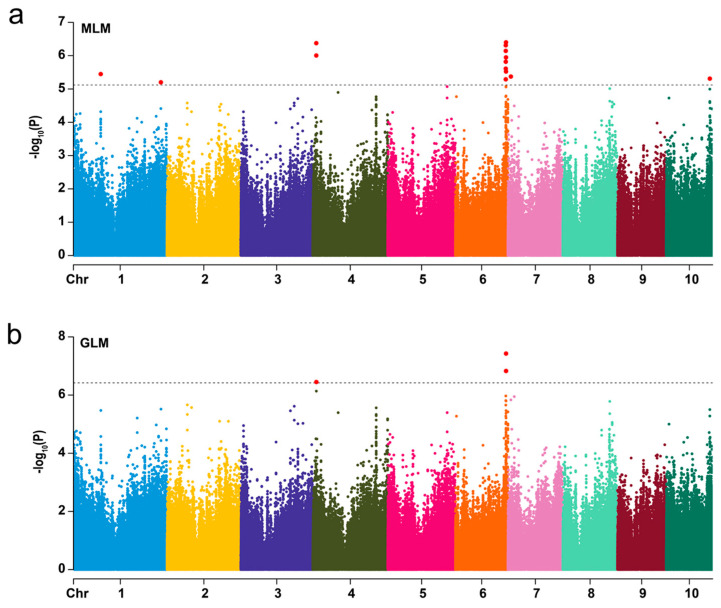
Manhattan plot for genome-wide association analysis of husk number. The SNPs are represented by dots. The horizontal dashed line represents the −log_10_(*P*) > 5.12 (*P* < 7.6 × 10^−6^) (**a**) and −log_10_(*P*) > 6.42 (*P* < 3.8 × 10^−7^) (**b**) significant thresholds, respectively. Red dots above the dashed line indicated SNPs significantly associated with husk number. MLM: mixed linear model; GLM: general linear model.

To reduce the effect of environmental variation, BLUP values across three environments (SY2015, TA2016, and TA2017) were used for the association study. The mixed linear model (MLM) was used to conduct GWAS for mining the significant SNPs associated with HN. Both population structure (Q matrix) and kinship relationship (K matrix) were incorporated to avoid false associations. With a threshold of −log_10_(*P*) > 5.12 (*P* < 7.6 × 10^−6^), a total of 16 significantly associated SNP sites were detected, which were distributed on chromosomes 1, 4, 6, 7, and 10 (Figure 2a, Table S2). These significant SNPs explained between 9.6% to 11.9% of the observed phenotypic variation. Furthermore, five SNPs were detected on chromosomes 4 and 6 at the threshold of −log_10_(*P*) > 6.42 (*P* < 3.8 × 10^−7^) by the general linear model (GLM), which could explain 7.8–10.4% of the phenotypic variation (Figure 2b, Table S2). A total of 16 SNPs were identified by the GLM and MLM, of which five SNPs were present in both models. When multiple significant SNPs were highly linked within a linkage disequilibrium (LD) block, they were merged into one genomic locus. We selected the peak SNP within each LD block for a subsequent analysis.

**Figure 3:**
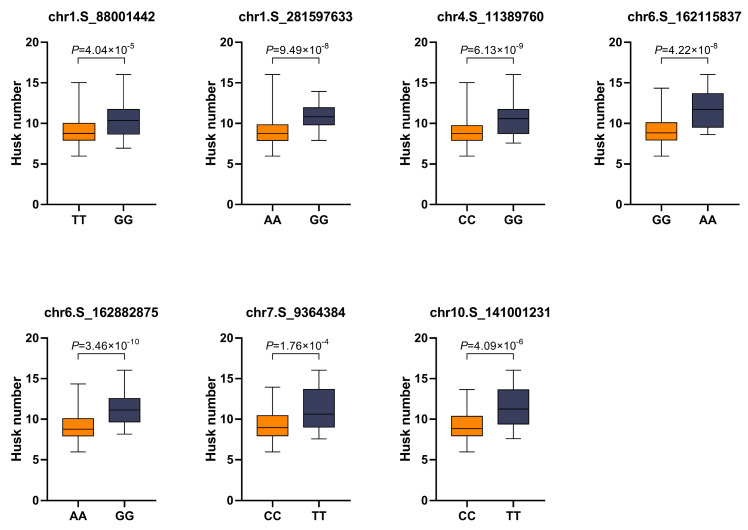
Allelic effects of the significant sites associated with husk number.

In this study, SNP alleles with a lower HN were defined as favorable alleles, whereas those with a higher HN were defined as unfavorable alleles. The allelic effects of different haplotypes of each significant SNPs on the HN trait were analyzed (Figure 3). The average HN for allele T of chr1.S_88001442 was 9.16, which was significantly smaller than that for allele C (10.39, *p* ≤ 0.001). Similarly, other favorable alleles for the HN included allele A of chr1.S_281597633, allele C of chr4.S_11389760, allele G of chr6.S_162115837, allele A of chr6.S_162882875, allele C of chr7.S_9364384, and allele C of chr10.S_141001231. The HN for lines carrying these alleles was 1.85, 1.50, 2.56, 2.08, 1.89, and 2.28 lower than those carrying unfavorable alleles, respectively.

**Figure 4:**
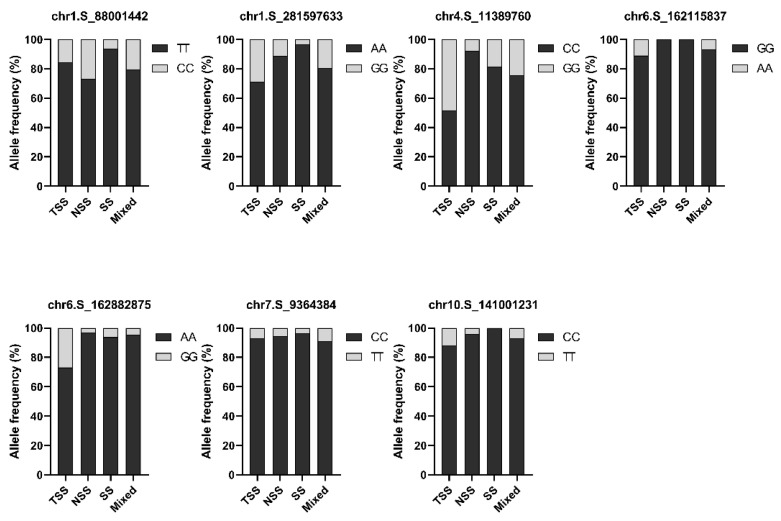
Allele frequency of SNPs significantly associated with husk number in different subpopulations. Dark-colored columns represent less-HN allele, and light-colored columns represent high-HN allele. TST, tropical/subtropical; SS, stiff-stalk; NSS, non-stiff-stalk; Mixed, the remaining inbred lines being Mixed subpopulation.

Further, we examined the allele frequency of significantly associated SNP sites in different subpopulations. Interestingly, the temperate maize inbred lines (SS and NSS) had higher allele frequencies for low-HN alleles for the most of significant SNPs, whereas tropical maize inbred lines (TST subpopulation) tended to favor the high-HN alleles (Figure 4). For example, for chr6.S_162115837, the high-HN allele (AA) is found in the TST subpopulation at a frequency of 87.80%, while it was zero in the SS and NSS subpopulations with the remaining 23.33% found in the Mixed population. Similarly, for chr4.S_11389760, the high-HN allele (GG) was observed with a frequency of 70.21% in the TST subpopulation, but its frequency was 11.34% in the SS and NSS subpopulations (Figure 4). These results suggest that the low-HN trait is favored in the temperate germplasms and may play a role during maize domestication and adaptation, leading to a rapid rise in the low-HN alleles within temperate maize germplasm.

### 2.3. Candidate Genes Related to the HN

Among the 16 SNPs that were detected by the MLM and GLM, 14 were present in the genic regions (Table S3). The SNP chr1.S_88001442 is located in the 5′UTR of *GRMZM2G071172*, and chr4.S_11389625 and chr4.S_11389760 are located in the fourth exon of *GRMZM2G008259*. chr6.S_162115732, chr6.S_162115778, chr6.S_162115837, chr6.S_162115848 and chr6.S_162115911 are located in the 5′UTR of *GRMZM2G175089*. chr6.S_162882875, chr6.S_162882956 and chr6.S_162883019 are located in the first exon of *GRMZM2G038032*, and chr6.S_162883376 is located in the intron of this gene. chr7.S_9364384 is located in the 18th intron of *GRMZM2G456059*. chr1.S_281597633 is located in the intergenic region, and the nearest gene is *GRMZM2G028151*. In addition, we calculated the LD between the associated SNP and neighboring SNPs within a 200 kb region (100 kb each upstream and downstream) to substantiate the evidence for identifying the candidate region (Figure S2). The genes located within the LD block around significant SNPs were considered as candidate genes. Finally, nineteen candidate genes have been located on chromosomes 1, 4, 6, 7, and 10 (3, 1, 10, 4, and 1 gene, respectively).

**Figure 5:**
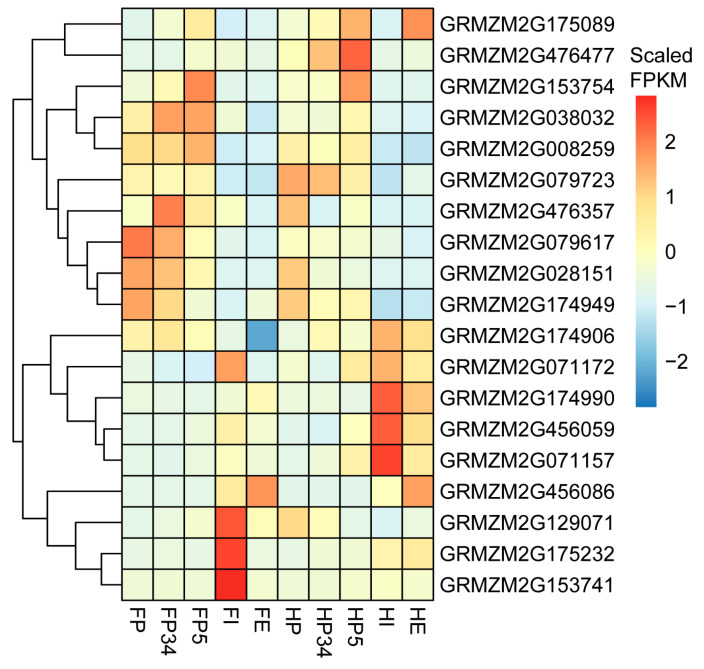
Heat-map of expression levels of the candidate genes identified by GWAS. The expression data are from leaf and husk leaf, and the description of the tissue is detailed in the study of Wang et al. [2]. Foliar primordia (FP), Foliar immature (FI), and foliar expanded (FE) leaf blades; husk primordia (HP), the outermost leaf on the ear (HE), and the third leaf in from the outside (HI). The color scale represents scaled FPKM values normalized using the z-score approach.

The candidate genes were divided into six functional types, including transcriptional regulation, signal transduction, metabolic, cellular transport, cell cycle regulation, and programmed cell death (Table S3). Based on published transcriptome data, the expression patterns of candidate genes were analyzed at different stages (including the primordium initiation period) of husk and leaf development (Figure 5). Except for *GRMZM2G476357*, whose expression is extremely low, the other genes are expressed in both the husk and leaf tissue. Specifically, *GRMZM2G038032*, *GRMZM2G174990*, and *GRMZM2G008259* are highly expressed in both tissues. The expression of *GRMZM2G038032* was higher in the early stage of primordium development, suggesting that this gene may regulate husk development at the critical early stage. In addition, nucleotide diversity analysis was performed on the *GRMZM2G038032* gene region and 5 kb upstream and downstream of the coding region using the third-generation Zea mays haplotype map (HapMap 3) data including teosintes, landraces, and modern maize varieties (Figure S3). The results showed that the nucleotide diversity of this gene’s promoter region in modern maize varieties was lower than that in teosinte and landraces, suggesting that the promoter/regulatory region of *GRMZM2G038032* was affected by selection during maize domestication. *GRMZM2G153754*, *GRMZM2G008259*, *GRMZM2G079723, GRMZM2G079617*, *GRMZM2G028151*, and *GRMZM2G174949* had similar temporal expression patterns to that of *GRMZM2G038032*: the expression levels of these genes were all higher at early stage of primordium development (Figure 5).

## 3. Discussion

### 3.1. Phenotypic Analysis of Husk Number (HN)

The maize husks wrap around the ear to help the normal growth and development of the kernel. However, excessive husk wrapping can significantly hinder grain dehydration after physiological maturity. The HN is a quantitative trait controlled by multiple genes or loci [11,23]. In this study, the HN of 232 maize inbred lines exhibited wide phenotypic variation and normal distribution. Maize originated in the tropics and subsequently adapted to the high latitudes of the temperate regions [24]. Comparing the HN among different subpopulations, our results indicate that maize from tropical/subtropical regions tends to have a higher husk number (Figure S1). The results of our study are consistent with those presented by Cui et al., who also showed that the rapid accumulation of fewer, narrower, and thinner husks in temperate maize germplasm as maize cultivation spreads from tropical to temperate regions [11].

### 3.2. SNP Sites Associated with HN via GWAS

Compared with linkage analysis, association analysis includes a wider range of natural variants and has the advantage of mining for more favorable allelic variants at higher resolution in a shorter period [25,26]. Maize is a model plant for association analysis because of its rich phenotypic variation and genetic diversity [20,27]. Currently, association analysis has become an efficient and powerful method to analyze the genetic mechanism of maize complex traits. In this study, we identified 16 SNPs significantly associated with the HN trait via GWAS. The favorable allelic variation in the identified SNPs had a significant regulatory effect on the HN (Figure 3), indicating that these SNPs had great potential application. These novel SNPs provide novel genetic loci for further analysis of the regulation mechanism of the HN in maize.

Previous studies have analyzed the correlations between husk number and other agronomic traits. The results showed that husk number was significantly correlated with the number of leaves above the ear, number of tassel branches, and flowering time-related traits, as well as some other traits [11]. In this study, to explore whether SNPs related to HN affect other agronomic traits, we analyzed the correlation between specific loci and eight traits using data sets from previous studies [28] (Figure S4). *tasselsheath1* (*tsh1*) and *tsh4* have been proven to establish developmental boundaries and maintain meristems, and mutants of these genes showed a reduced tassel branch and increased HN [29,30]. In this study, the SNPs chr4.S_11389760 and chr6.S_162882875 significantly affected tassel branch number (TBN) (Figure S4d), suggesting that HN and TBN may be subject to similar genetic regulation mechanisms, although these two traits were positively correlated. The leaf number above ear (LNE) at chr6.S_162115837 and chr6.S_162882875 was also positively correlated with the HN (Figure S4e). In addition, lines carrying low-HN alleles flowered significantly earlier than those with high-HN alleles (Figure S4a–c). Previous research has found that photoperiodic response factor *ZmELF3.1* regulates gene expression of tsh4 by forming a protein complex with RAMOSA2 (RA2) [31]. These HN-related SNPs did not affect yield traits, including panicle length, row number, and 100-grain weight (Figure S4f–h). The above evidence shows the correlation between the HN and other traits. It is of great significance to understand the potential similar genetic mechanism between different traits and consider breeding for multiple traits.

### 3.3. Putative HN-Related Genes

The genes related to ear development and their regulatory pathways have been extensively studied. However, the molecular mechanism of the development of husk, which is a protective organ for healthy ear growth, is poorly understood. At present, only one gene, *ZMET2*, which regulates the HN, has been cloned [21]. Most of the existing studies remain at the preliminary stage, including large QTL intervals and SNP loci identified by GWAS and linkage analysis, and key genes at these loci have not been verified. In this study, several novel SNP sites associated with HN were identified via GWAS. We extracted genes within the LD block around peak SNPs and identified 19 candidate genes with functional annotations. According to the functional annotation, these genes are primarily categorized into several functional groups: transcriptional regulation, signal transduction, and metabolic. These processes are essential for plant growth and organ development.

Transcription factors are a class of important regulatory proteins, which is involved in multiple processes of plant growth and development. In this study, we identified five transcription factors *GRMZM2G028151*, *GRMZM2G008259*, *GRMZM2G175232*, *GRMZM2G476357*, and *GRMZM2G153754*. Specifically, *GRMZM2G028151* encodes AP2/EREBP protein. AP2/EREBP family regulates many biological processes in plants, such as plant morphogenesis, hormone signal transduction, and the regulation of the metabolic process. The loss of AP2 function has been shown to cause the homeotic transition of sepals to carpels, and petals to stamens, as *AP2-5*, *AP2-6*, and *AP2-7* mutants in Arabidopsis [32,33]. The maize *branched silkess1* (*bd1*) of the AP2/ERF family was specifically expressed in the ear, which affects flower organ development [34]. The AP2-like genes played an important role in modifying spike characteristics in barley and wheat [35]. Thus, it is speculated that the husk developed from the axillary meristem may also be regulated by the AP2 family gene. The MYB family is one of the largest transcription factors families in plants. *GRMZM2G175232* encodes MYB99 in maize. In Arabidopsis, several MYB transcription factors such as *AtMYB37/RA1*, *AtMYB38*/*RAX2/BIT1*, *AtMYB84/RAX3*, *AtMYB105/LOF2*, and *AtMYB117/LOF1* have been shown to be regulators of axillary meristem formation [36,37,38]. In addition, *AtMYB59* and *AtMYB77* have been confirmed to regulate root development and lateral root formation, respectively [39,40]. These studies indicate that MYB transcription factors play an important role in plant organ development.

Proper plant growth and development require various metabolites such as hormones, lipids, vitamins, and amino acids, and the synthesis and regulation of these substances are integral components of the plant metabolic pathway [41,42]. Plant metabolic pathways are complex and usually involve multiple enzymes. We found that candidate genes include several related enzymes in different metabolic pathways, including glucosaminly transferase (*GRMZM2G071172*), Polygalacturonase (*GRMZM2G079617*), and ribose-5-phosphate isomerase (*GRMZM2G456086*). Among them, Polygalacturonase is involved in cell expansion and division, and influences cell wall dynamics by regulating the structure of the cell wall [43].

How plants regulate and determine their growth, development, and morphogenesis is inseparable from complex signal transduction processes. Eight genes related to cell signal transduction were identified in this study. We identified a Rho of plants (ROP) guanine-nucleotide exchange factor (GEF), *GRMZM2G071157*. ROP is a key protein for polar signal transduction and controls auxin-dependent polar cell elongation during plant development [44]. *ROPGEF1* was shown to regulate the polarization of the auxin influx carrier AUX1 and the accumulation of efflux carriers PIN7 and PIN2 [45]. Mutations in *ROPGEF1* and *ROPGEF7* result in embryonic defects and abnormal cotyledon development [45,46]. Protein kinases control a series of cell processes, including metabolism, transcription, and cell cycle [47]. *GRMZM2G456059* encodes a serine/threonine-protein kinase, which is one of the major protein kinases.

*GRMZM2G038032* encodes a receptor for activated C kinase 1 (RACK1). As a scaffold protein, RACK1 regulates plant growth and development by connecting various plant hormone signaling pathways [48], and its expression can be induced by auxin and cytokinin [49]. RACK1 has been demonstrated to interact with *GIF1* (GRF-interacting factor1), a key gene that regulates hormone biosynthesis and meristem determination and controls the structure and morphological development of maize ear [50]. *GIF1* mutants frequently develop axillary shoots in the axils of husk leaves and have an increased husk number [51]. In Arabidopsis, *GIF1*/*AN3* is necessary for the maintenance of shoot apical meristem, and is involved in the regulation of cell proliferation, growth, and lateral organ growth [52,53,54]. As *GRMZM2G038032* was highly expressed in the husk primordium (Figure 5), we postulate that this gene may be involved in the regulation of husk primordia developed from the axillary meristem through interactions with GIF1 in maize. In addition, through a nucleotide polymorphism analysis, we found that the sequence diversity of the promoter region of *GRMZM2G038032* in modern maize is low, indicating that this region was selected during domestication (Figure S3). Hence, it is reasonable to speculate that *GRMZM2G038032* is the most likely candidate gene on chromosome 6 for the regulation of HN in maize.

In summary, by analyzing the functional annotation and expression level of candidate genes, we speculated the possible pathways of some candidate genes controlling the HN trait. The other candidate genes that have not been discussed may also be involved in husk development in currently unknown regulatory pathways.

## 4. Materials and Methods

### 4.1. Plant Materials and Field Experiment

GWAS was conducted on a diverse panel comprising 232 maize inbred lines, which included tropical/subtropical (TST), temperate (SS, NSS), and mixed germplasms, as detailed in Table S1. This association panel was cultivated in three environments in China: Sanya, Hainan Province (SY, 18.22° N, 109.01° E) in November 2015, and Taian, Shandong Province (TA, 36.09° N, 117.09° E) in May 2016, and in May 2017. The husk number (HN) of 230, 222, and 188 inbred lines were counted in the three environments, respectively. Each inbred line was planted with seven plants per row, the and row spacing and plant spacing were about 0.6 and 0.2 m, respectively. Field management during the entire growing period was the same as local field management practices.

### 4.2. Phenotyping and Statistical Analysis

After the ears matured, the plants with grow well and uniformly in each inbred line were harvested to count the HN. Phenotypic data were represented by the mean values of the selected three to five ears. The Microsoft Excel 2010 software package was used to organize phenotypic data and conduct descriptive statistical analysis. Phenotypic variation in the husk number trait was performed using the lme4 package of the R 4.0.1 software program. The ANOVA model is as follows: y_ij_ = μ + g_i_ + e_j_ + ε_ij_, where y_ij_ is the phenotypic value of the husk number, μ is the mean value across environments, g_i_ is the effect of the genotype, e_j_ is the effect of the environment, and ε_ij_ is the residual error. The broad-sense heritability (*H*^2^) was calculated as follows: $H^{2}=\frac{\sigma_{g}^{2}}{\sigma_{g}^{2}+(\sigma_{gy}^{2}/n)}$ [55], where $\sigma_{g}^{2}$ is genetic variance, $\sigma_{gy}^{2}$ is the residual variance, and n is the number of environments. To minimize environmental effects, the best linear unbiased prediction (BLUP) values for the husk number across all environments were estimated for each line using the same ANOVA model.

### 4.3. Genome-Wide Association Analysis

The genotype data used in this association panel were detailed in a prior study by Liu et al. [56]. The genotype data included 1.25 million SNPs with minor allele frequency (MAF) ≥ 0.05 and consisted of four sets (MaizeSNP50 BeadChip, 600K SNP array, RNA-seq, and genotyping by sequencing (GBS)), and the data can be downloaded at http://www.maizego.org/Resources.html (accessed on 26 March 2025). In addition, we retained only bi-allelic SNPs with a missing data rate less than 20% within this association panel. A total of 995,106 SNPs were ultimately retained to conduct GWAS. The GWAS for HN was performed using TASSEL (v5.0) [57] (Bradbury et al., 2007) under the mixed linear model (MLM) and the general linear model (GLM). The MLM was conducted using population structure (Q matrix) and kinship relationship (K matrix) to avoid spurious associations [58,59]. The SNPs in the GWAS are not independent due to the linkage disequilibrium (LD) among them. We therefore used the effective number of independent markers for the adjustment of multiple markers to obtain the P-value thresholds. A total of 131,432 markers in approximate linkage equilibrium (the LD R^2^ threshold is 0.2) were found by PLINK [60]. We used the uniform Bonferroni-corrected thresholds at α = 1 for the MLM and α = 0.05 for the GLM as the cut-offs as applied in previous studies [28,61,62]. Therefore, the suggestive *p*-value 7.61 × 10^−6^ and 3.8 × 10^−7^ were established for the MLM and GLM, respectively, to identify significant SNPs with the HN.

### 4.4. Annotation of Candidate Genes

We used the PLINK 1.9 package to calculate the LD between the associated SNP and neighboring SNPs within a 200 kb region (100 kb upstream and downstream) to identify the candidate region. Genes in the same LD block (r^2^ > 0.2) with significant SNPs were considered candidate genes. Functional annotation and prediction of candidate genes were performed through the NCBI (http://www.ncbi.nlm.nih.gov/) website and MaizeGDB database (http://www.maizegdb.org/). Transcriptome data were obtained from a published study by Wang et al. [2]. The third-generation *Zea mays* haplotype map (HapMap 3) data were downloaded from https://www.panzea.org/ and were used to examine the nucleotide diversity around *GRMZM2G038032* in maize and its wild relative, teosinte.

## 5. Conclusions

Based on the GWAS of the MLM and GLM, a total of 16 SNPs were associated with HN. The discovered candidate genes may co-regulate husk development in different pathways, among which *GRMZM2G038032* located on chromosome 6 is likely the most relevant gene, though further verification is needed. The identified SNPs related to the HN trait provide a reference set not only for marker-assisted selective breeding for low husk number and faster grain dehydration, but also for the prediction of candidate genes for unveiling the regulatory mechanism of husk development.

## Figures and Tables

**Table 1:** Descriptive statistical analysis of husk number traits in maize.

Environment	Range	Mean **±** SD	CV (%)	Skewness	Kurtosis	*H*^2^ (%) ^a^
SY2015	4.80–19.00	8.56 ± 2.14	25.35	1.15	2.01	88.62
TA2016	6.00–18.33	9.85 ± 2.25	22.84	1.04	1.33	
TA2017	5.50–16.75	9.70 ± 2.22	22.89	0.94	0.63	

SY2015: Sanya in 2015; TA2016: Taian in 2016; TA2017: Taian in 2017; SD: standard deviation; CV: coefficient of variation; ^a^ broad-sense heritability estimated across three environments.

## Data Availability

The original contributions presented in this study are included in the article/Supplementary Materials. Further inquiries can be directed to the corresponding authors.

## Supplementary Materials

The following supporting information can be downloaded at: https://www.mdpi.com/article/10.3390/ijms26073437/s1.
