# Supplementary material for: Genome-Wide Association Study and Candidate Gene Mining of Husk Number Trait in Maize
Source: Int J Mol Sci. 2025 Apr 7;26(7):3437. doi: 10.3390/ijms26073437 (PMC11989285; doi:10.3390/ijms26073437)
Supplement: Supplementary file 1 [file ijms-26-03437-s001.zip › Supplementary Figures.pdf]

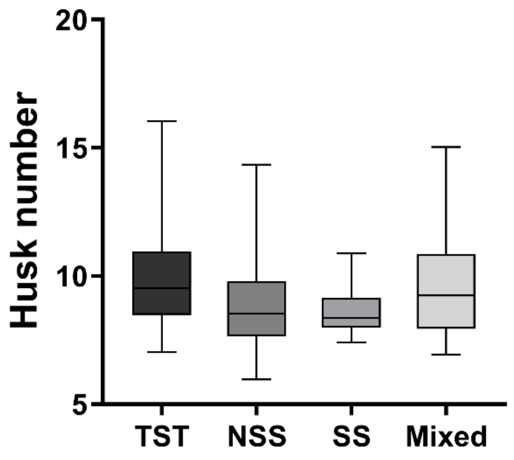

Figure S1. Comparative analysis of husk number in different subpopulations. TST, tropical/subtropical; SS, stiff-stalk; NSS, non-stiff-stalk; Mixed, the remaining inbred lines being Mixed subpopulation.

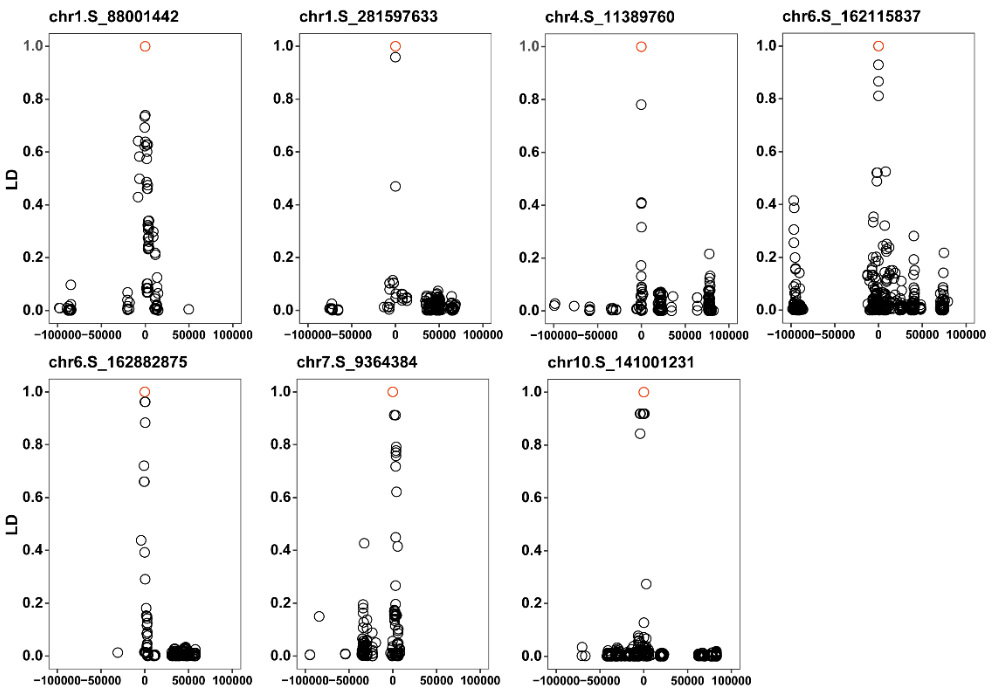

Figure S2. Linkage disequilibrium (LD) decay within 200 kb surrounding the significant SNPs. The physical position of seven significant SNPs (red circles) associated with HN were defined as zero in x axis.

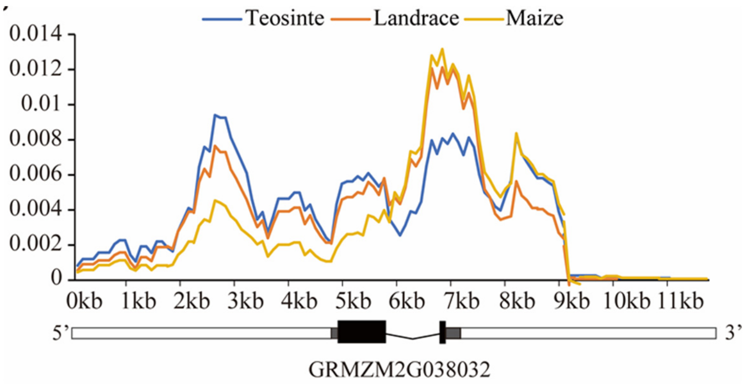

Figure S3. Analysis of nucleotide diversity of *GRMZM2G038032* gene region and 5 kb sequence of gene upstream and downstream in teosinte, landrace and maize.

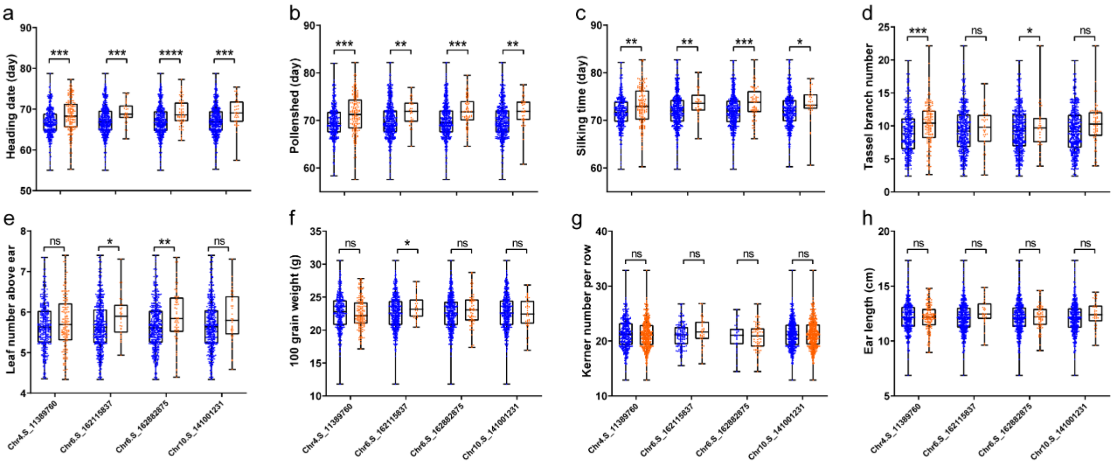

Figure S4. Effects of HN-related SNP loci on other agronomic traits. Phenotypic comparison of eight agronomic traits between inbred lines carrying low-HN alleles and high-HN alleles at four SNP loci. The blue dots represent lines of low-HN alleles, orange dots represent lines of high-HN alleles.
